# Colorectal Cancer in Young Adults in Panama: Clinical, Molecular, and Epidemiological Characterization in a National Cohort

**DOI:** 10.7759/cureus.100580

**Published:** 2026-01-01

**Authors:** Jose Pinto Llerena

**Affiliations:** 1 Oncology, Hospital Anita Moreno, Chitré, PAN

**Keywords:** colorectal cancer, early-onset colorectal cancer, latin america, prognosis, survival analysis, young adults

## Abstract

Background: Early-onset colorectal cancer (≤49 years) is rising worldwide, yet data from Central America are scarce. We characterized clinical, molecular, and therapeutic features, estimated overall survival, and identified independent prognostic factors in young adults with colorectal cancer in Panama.

Methods: We used a retrospective cohort (2020-2023) from two national oncology referral hospitals. Demographic, clinical, molecular (RAS, microsatellite instability-high), behavioral (tobacco, alcohol, body mass index), and treatment data were collected. Overall survival was estimated using the Kaplan-Meier method; multivariable Cox regression assessed prognostic factors. The statistical software Stata version 18 (StataCorp, College Station, TX, USA) was used for data analysis.

Results: We included 242 patients (median age 44 years), 126 (52%) of whom were female. Stage III-IV comprised 172 patients (71%), and stage IV comprised 103 patients (47%). Rectal tumors accounted for 83 patients (35%). RAS mutation was detected in 63 patients (36% overall and 61.3% of evaluable stage IV cases) and microsatellite instability-high in 19 patients (7.8%). Risk factors included tobacco use in 31 patients (12.8%), alcohol intake in 73 patients (32%), and overweight/obesity in 108 patients (44%). The geographical areas with the highest population density in the country have the highest case percentages, such as the province of Panama, with 105 patients (43.7%). Median overall survival was 34 months; overall survival at one, three, and five years was 78%, 49%, and 32% (95% CI 19.4-47.0). In multivariable analysis, independent predictors of worse overall survival were stage IV at diagnosis (p=0.001), baseline Eastern Cooperative Oncology Group (ECOG) score ≥2 (p=0.008), poor differentiation (p=0.020), and recurrence (p=0.030).

Conclusions: Early-onset colorectal cancer in Panama presents predominantly at advanced stages and is associated with limited survival. Stage, functional status, histologic differentiation, and recurrence independently predict mortality. Findings underscore the need for earlier detection, access to molecular testing, and equitable delivery of modern therapies.

## Introduction

Colorectal cancer (CRC) is the third most common malignancy and the second leading cause of cancer-related death globally [1,2]. Over the past two decades, multiple regions have reported a sustained rise in early-onset CRC (≤49 years), including North America, Europe, and parts of Latin America [3]. Young adults frequently present with locally advanced or metastatic disease, distal and rectal primaries, and adverse histologies such as mucinous and signet-ring cell carcinoma; these features translate into a disproportionate burden of treatment, productivity loss, and premature mortality [4,5].

Despite increasing awareness, evidence from Central America and the Caribbean remains limited, hampering region-specific policies for screening and care delivery. Latin American cohorts suggest five‑year survival rates around 30-35% across all stages, lower than those reported in high‑income settings, and highlight inequalities in access to specialist care, biologic agents, and immunotherapies [5,6]. Risk factor profiles, including tobacco and alcohol exposure and the growing prevalence of overweight/obesity, may interact with tumor biology to shape outcomes in early-onset CRC [7-9]. At the molecular level, RAS pathway alterations and mismatch repair deficiency have immediate therapeutic implications, informing use of anti‑EGFR agents and immune checkpoint inhibitors, respectively [10-13].

In this context, we report the first nationwide cohort of early-onset CRC from Panama. We describe clinical and molecular characteristics, quantify survival, analyze independent prognostic factors, and estimate prevalence by province using the most recent census denominators. We also compare findings with international and regional series to contextualize public health needs [9].

## Materials and methods

Study design and setting

We conducted a retrospective cohort study at Panama’s two public national oncology referral hospitals, including patients diagnosed between January 2020 and December 2023. Data were collected from the digitized medical records of patients in the electronic health record system of the National Oncology Network of Panama.

Eligibility criteria

Inclusion criteria were age ≤49 years and histologically confirmed CRC. We excluded records lacking essential dates (diagnosis or follow‑up).

**Figure 1:**
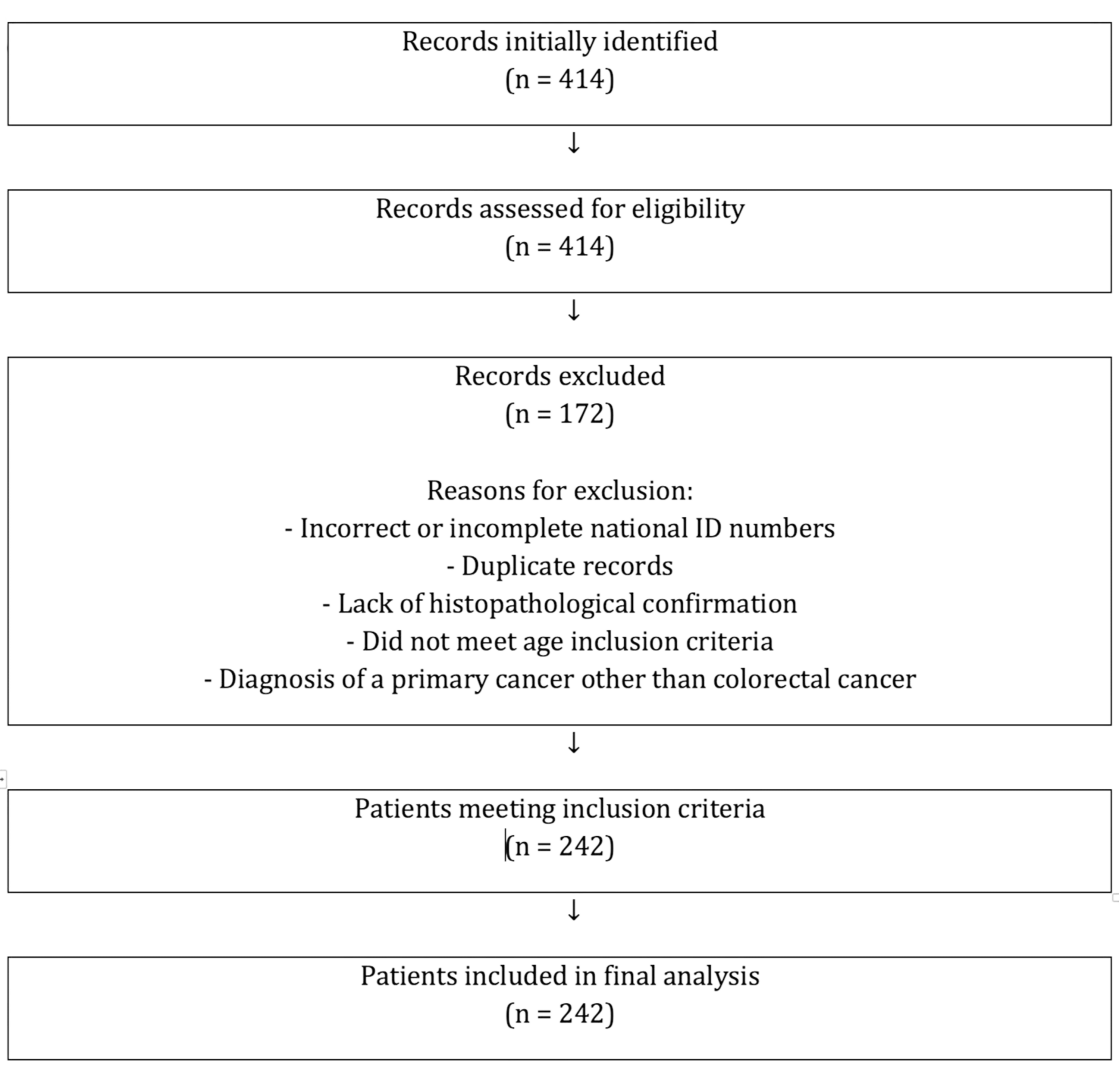
Strengthening the Reporting of Observational Studies in Epidemiology (STROBE) Flow Diagram of Patient Selection

The patient selection process is summarized in a flow diagram in accordance with Strengthening the Reporting of Observational Studies in Epidemiology (STROBE) guidelines (Figure 1). A total of 414 subjects were initially identified. After eligibility assessment, 170 records were excluded due to incorrect or incomplete national identification numbers, duplicate entries, lack of histopathological confirmation, failure to meet age inclusion criteria, or confirmation of a primary malignancy other than CRC. Ultimately, 244 patients met the inclusion criteria and were included in the final analysis.

Variables

Demographics (age, sex, province), clinical (American Joint Committee on Cancer (AJCC) 8th stage, Eastern Cooperative Oncology Group (ECOG), primary site, histology, grade), molecular (RAS status, microsatellite instability (MSI) status), behavioral/comorbid (tobacco, alcohol, BMI category, hypertension, diabetes), and treatments (surgery, adjuvant therapy, palliative systemic therapy, biologics, radiotherapy, immunotherapy for MSI-high (MSI-H) candidates).

Outcomes

The primary outcome was overall survival (OS), defined as the time from first intervention to death from any cause or last follow-up. Vital status was coded as 1 = dead and 2 = alive.

Statistical analysis

Baseline characteristics were summarized with medians/IQRs and proportions. OS was estimated using the Kaplan-Meier method and compared by log‑rank test. Multivariable Cox proportional hazards models assessed independent prognostic factors. A parsimonious model was constructed prioritizing variables with clinical relevance and acceptable completeness (stage, ECOG, grade, recurrence, sex, age group, histology, laterality, RAS). Results are presented as hazard ratios (HR) with 95% confidence intervals. Analyses were conducted using standard statistical software STATA version 18 (StataCorp, College Station, TX, USA).

Ethics

The protocol was approved by the institutional bioethics committee and conducted in accordance with the Declaration of Helsinki; patient confidentiality was preserved. 

## Results

Baseline characteristics (n=242)

Median age was 41 years (IQR 35-45), and 126 (52.1%) were women, an uncommon female predominance compared with most international early-onset CRC series. Sixty‑two percent presented with stage III-IV disease, with stage IV being the most frequent, with 103 (47%) cases. Rectal tumors accounted for 83 (35%) cases, sigmoid for 63 (26.7%), ascending colon for 22 (9.3%), transverse for 22 (9.3%), cecum for 21 (8.9%), descendent for 13 (5.4%) and rectosigmoid junction for 13 (5.4%); adenocarcinoma not otherwise specified (NOS) predominated with 198 (82%) cases, followed by mucinous with 21 cases (11%), and signet‑ring with 13 cases (4%). Eight cases were other histologies (3%). ECOG performance status was 1 in 153 (63%). Comorbidities were present in 80 (33%), mainly hypertension and diabetes. Baseline clinical and epidemiological characteristics are summarized in Table 1. 

**Table 1:** Clinical-epidemiological characteristics and risk factors ECOG: Eastern Cooperative Oncology Group, NOS: not otherwise specified, CRC: colorectal cancer

Variable	Frequency (n)	Percentage (%)
Age group <20	3	1.2
20–30	21	8.7
31–40	61	25.2
41–49	157	64.9
Median age (years)	44 (16–49)	—
Female sex	126	52.1
ECOG <2	224	92.6
ECOG ≥2	18	7.4
Any comorbidity	80	33.1
Stage I–II	46	21.1
Stage III–IV	172	71.1
Overweight/Obesity	108	44.6
Smoking use	31	12.8
Alcohol use	73	30.2
Histology: Adenocarcinoma NOS	198	82.0
Family history of cancer	46	19.0
Family history of CRC	27	11.0
Primary site: Rectum	83	35.0

**Figure 2:**
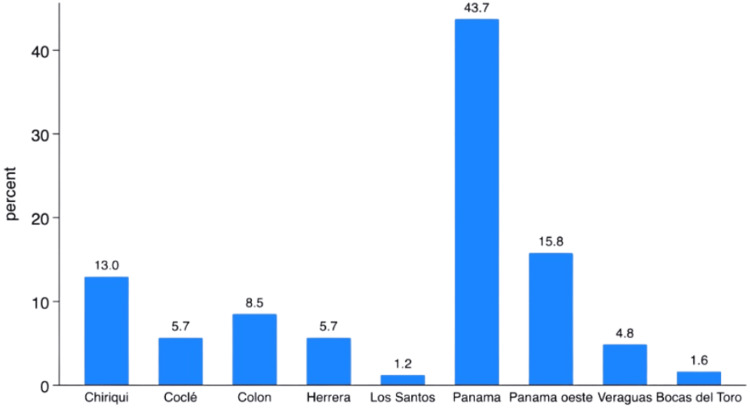
Distribution of cases by province in Panama

The distribution of cases by province indicated that the majority of cases are diagnosed in areas with the highest young population density in the country: Panama City with 105 (43.7%), Panama Oeste with 36 (15.8%), and Chiriqui with 32 (13%). The remaining information is detailed in Figure 2.

Molecular and behavioral features

RAS mutation was detected in 63 (36%) patients; among evaluable stage IV patients, 63 (61.3%) were RAS‑mutant. MSI-H was documented in 19 patients (7.8%), with loss of MLH1 plus PMS2 in 11 patients, who were considered for immunotherapy based on clinical context. Regarding risk factors, 31 patients (12.8%) reported current or past tobacco use, 73 patients (30%) had significant alcohol intake, and 108 patients (44.6%) were overweight or obese. 

Therapies

Surgery with curative intent was performed in 147 patients (60.7%), chemotherapy in 241 patients (99.6%), 134 patients (55%) in adjuvant scenario, palliative systemic therapy in 159 patients (65%), biologics in 34 patients (14%), and radiotherapy in 79 patients (32%), predominantly for rectal tumors. 

Metastatic sites

Among patients with stage IV disease or recurrence, the peritoneum was the most common site of metastasis with 51 cases (21%), followed by the liver with 31 cases (12.8%) and the lung with eight cases (3.3%). Other distant sites (e.g., distant lymph nodes, bone) were rare. Table 2 shows the treatment modalities, metastases, and molecular profile of the cohort.

**Table 2:** Treatments, molecular profile, recurrence, and metastasis Percentages may not total 100% due to rounding. Missing data included as reported. Note: BRAF mutation detected in three patients (1.2%). RAS: rat sarcoma viral oncogene homolog, MSI-H: microsatellite instability–high

Variable	Frequency (n)	Percentage (%)
Surgery		
Yes	147	60.7
No	95	39.3
Chemotherapy		
Yes	241	99.6
No	1	0.4
Radiotherapy		
Yes	79	32.6
No	163	67.4
Recurrence		
Yes	43	17.8
No	91	37.6
Metastasis		
Single site (peritoneum/liver/lung)	90	37.2
Multiple sites	42	17.4
Others	13	5.4
RAS status		
Mutated	63	26.0
Wild-type	50	20.7
MSI-H status		
Present	19	7.9
Absent	47	19.4
Missing	176	72.7

Survival

**Figure 3:**
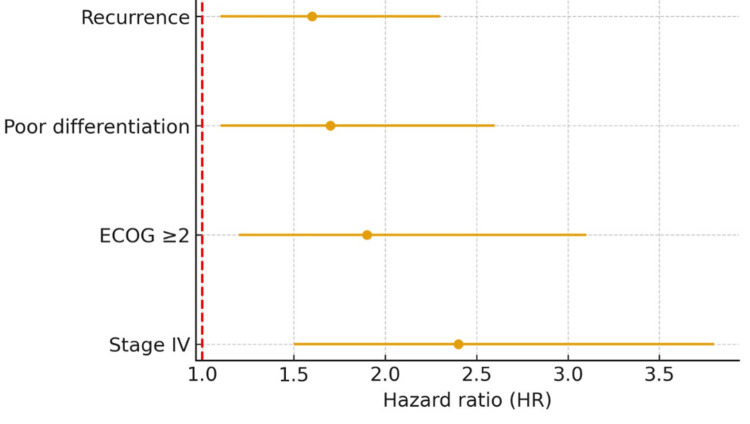
Forest plot of independent prognostic factors for overall survival (multivariable Cox model). ECOG: Eastern Cooperative Oncology Group

**Figure 4:**
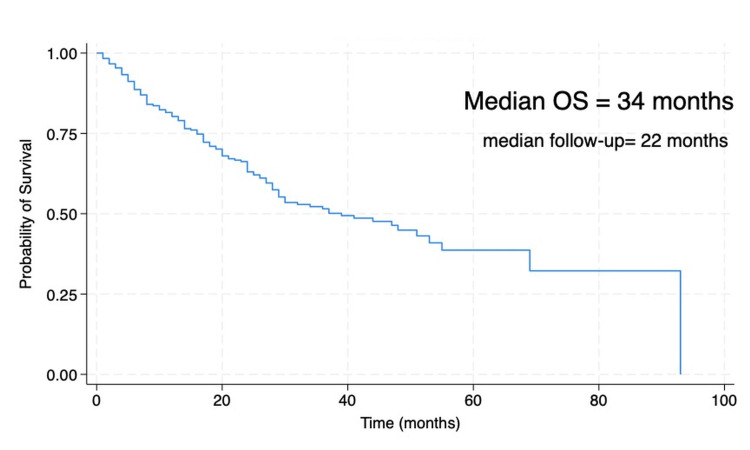
Kaplan–Meier overall survival (OS)

The median OS was 34 months. OS at one year, three years, and five years was 78%, 49%, and 32% (95% CI 19.4-47.0), respectively. The median follow-up was 22 months, indicating that half of the patients were observed for nearly two years after diagnosis. This follow-up duration ensures sufficient reliability for the OS estimates reported in this study. In multivariable Cox models, independent predictors of worse OS included stage IV at diagnosis (HR 2.4, 95% CI 1.5-3.8, p=0.001), ECOG ≥2 (HR 1.9, 95% CI 1.2-3.1, p=0.008), poor differentiation (HR 1.7, 95% CI 1.1-2.6, p=0.020), and recurrence (HR 1.6, 95% CI 1.1-2.3, p=0.030). Independent prognostic factors identified in the multivariate analysis are detailed in Table 3 and the Forest plot in Figure 3. Survival is depicted in Figure 4. 

**Table 3:** Multivariable Cox regression: independent prognostic factors for overall survival (OS) ECOG: Eastern Cooperative Oncology Group, BMI: Body Mass Index, RASwt: rat sarcoma viral oncogene homolog wild-type

Variable	HR	95% CI	p-value
Stage IV	2.4	1.5–3.8	0.001
ECOG ≥2	1.9	1.2–3.1	0.008
Poor differentiation	1.7	1.1–2.6	0.020
Recurrence	1.6	1.1–2.3	0.030
Age	0.99	0.9-1.0	0.704
Sex	1.05	0.5-1.9	0.871
Histology type	0.64	0.3-1.3	0.230
BMI	1.03	0.7-1.3	0.825
Laterality	1.27	0.4-1.4	0.648
RASwt	0.86	0.4-1.4	0.594

## Discussion

This nationwide cohort shows that Panamanian young adults with CRC are frequently diagnosed at advanced stages and experience limited survival (median OS 34 months; five‑year OS 32%). Independent prognostic factors - stage IV, ECOG ≥2, poor differentiation, and recurrence - are consistent with established literature and emphasize the dual importance of disease biology and host fitness [10,11]. 

Most patients presented with good performance status with an ECOG score of <2 in 224 patients (92.6%), few comorbidities in 80 patients (33%), and a low prevalence of smoking in 31 patients (12.8%) and alcohol consumption in 73 patients (30.2%). However, a high prevalence of overweight and obesity was observed, affecting 108 patients (44.6%). A family history of cancer was present in only 46 patients (19%), and only 27 patients (11%) had a specific family history of CRC. These findings are consistent with those reported by Ruíz et al. and Akimoto et al. [14,15].

Curative-intent surgery was attempted in 147 patients (60.7%); however, 159 patients (65%) ultimately required palliative systemic therapy. This pattern likely reflects the high rate of advanced disease at diagnosis, with 172 patients (71.1%) presenting with stage III or IV disease, which may explain this characteristic of our population and the established literature [10].

Two findings merit particular emphasis. First, a slight female predominance of 126 patients (52%) contrasts with many international series where early-onset CRC is more common in men [8]; this could reflect regional differences in healthcare‑seeking behavior or risk factor profiles and warrants further study. Second, peritoneal metastasis was the leading metastatic site (51 cases), surpassing the liver and lung. This pattern, less typical in unselected CRC cohorts, could be related to the higher frequency of mucinous and signet‑ring histologies observed in young patients, both of which are associated with peritoneal dissemination [16]; these observations have pragmatic implications for staging, surveillance, and surgical/hyperthermic intraperitoneal chemotherapy (HIPEC) discussions in selected cases.

Molecularly, the proportion of RAS‑mutant tumors (36% overall; 61.3% in metastatic) and MSI-H (8.3%) underscores the necessity of universal testing to inform precision therapy, anti-EGFR antibodies in RAS-wild-type tumors, and checkpoint inhibitors for MSI-H disease. The implementation of these tests should be strengthened in oncologic care in Panama, as MSI-H status was not assessed in 176 patients (72.7%), despite its importance for therapeutic decision-making in CRC at that time and continuing to the present day. From a public health perspective, heterogeneity in case distribution, with Panama City markedly above the rest of the country, suggests geographic disparities in risk, detection, and/or registry capture, paralleling inequalities described in other Latin American contexts. Addressing these gaps will require early detection policies tailored to younger populations, streamlined referral pathways, and equitable access to modern systemic therapies [17-22].

Public health and policy implications in Latin America

The results of this study have clear implications for oncology care in low- and middle-income countries [23]. The high prevalence in provinces such as Herrera suggests disparities in risk factors, timely diagnosis, and continuity of care. In Panama, as in many Latin American countries, delayed diagnosis and limited access to molecular testing or targeted therapies contribute to advanced-stage presentation. Strengthening screening strategies for younger populations, expanding access to molecular diagnostics (RAS, MSI), and ensuring equitable access to systemic and immunotherapies are priorities [13].

This study reinforces findings shared by other Latin American countries, for example, in Chile, socioeconomic inequities strongly correlate with both stage at diagnosis and survival outcomes in CRC, particularly in the young-onset population [24]. Recent estimates for Latin America and the Caribbean project a significant increase in CRC incidence and mortality over the next few decades unless preventive and early detection measures are scaled up [24]. Early-onset CRC is characterized by distinct molecular features and a more aggressive clinical course compared to late-onset disease [25]. The Quick Comment highlights barriers to molecular testing and therapeutic access across multiple Latin American countries, which may contribute to worse outcomes in young CRC patients [26]. Time trends in CRC incidence in Costa Rica demonstrate increasing rates across multiple regions, particularly among younger adults [27]. 

These results align with Latin American studies showing similar challenges, reinforcing that structural inequities in health systems, rather than biology alone, drive poor outcomes in early-onset CRC [5,9,21].

Strengths include the first national early-onset CRC cohort from Central America with integrated clinical and molecular data, survival estimates extending to five years and beyond, and an appropriate design and clear theoretical grounding. Limitations include the retrospective design, missingness for some molecular variables, and reliance on information documented in the electronic medical records; no patients were directly interviewed, progression-free survival or disease-free survival were not analyzed, and there is potential referral bias. Nonetheless, our results provide actionable evidence to inform screening strategies, resource allocation, and future prospective multicenter studies in the region.

## Conclusions

Early-onset CRC in Panama is characterized by advanced‑stage presentation, substantial peritoneal metastatic burden, and limited survival. Stage, ECOG, histologic differentiation, and recurrence independently predict mortality. Scaling early detection, ensuring universal molecular testing, and improving access to evidence‑based therapies should be prioritized in national cancer control plans.
